# The short-sequence design of DNA and its involvement in the 3-D structure of the genome

**DOI:** 10.1038/s41598-018-35864-9

**Published:** 2018-12-13

**Authors:** Guillermo Lamolle, Victor Sabbia, Héctor Musto, Giorgio Bernardi

**Affiliations:** 1 Laboratorio de Organización y Evolución del Genoma, Unidad Genómica Evolutiva, Facultad de Ciencias, Montevideo, Uruguay; 2 Science Department, Roma Tre University, Viale Marconi 446, 00146 Rome, Italy; 3 Stazione Zoologica Anton Dohrn, Villa Comunale, 80121 Naples, Italy

## Abstract

Recent investigations have shown that isochores are characterized by a 3-D structure which is primarily responsible for the topology of chromatin domains. More precisely, an analysis of human chromosome 21 demonstrated that low-heterogeneity, GC-poor isochores are characterized by the presence of oligo-Adenines that are intrinsically stiff, curved and unfavorable for nucleosome binding. This leads to a structure of the corresponding chromatin domains, the Lamina Associated Domains, or LADs, which is well suited for interaction with the lamina. In contrast, the high-heterogeneity GC-rich isochores are in the form of compositional peaks and valleys characterized by increasing gradients of oligo-Guanines in the peaks and oligo-Adenines in the valleys that lead to increasing nucleosome depletions in the corresponding chromatin domains, the Topological Associating Domains, or TADs. These results encouraged us to investigate in detail the di- and tri-nucleotide profiles of 100 Kb segments of chromosome 21, as well as those of the di- to octa-Adenines and di- to octa-Guanines in some representative regions of the chromosome. The results obtained show that the 3-D structures of isochores and chromatin domains depend not only upon oligo-Adenines and oligo-Guanines but also, to a lower but definite extent, upon the majority of di- and tri-nucleotides. This conclusion has strong implications for the biological role of non-coding sequences.

## Introduction

The human genome (like other mammalian genomes) is compositionally compartmentalized into isochores, large (≥0.2 Mb), “fairly homogeneous” DNA sequences that belong to five families, L1, L2, H1, H2, H3, characterized by increasing GC ranges, increasing compositional heterogeneities, and increasing gene densities^1–3^ (see Supplementary Table S1 and Fig. S1 of refs^4^, and ^5^ for a recent review).

The functional importance of isochores was evident for a long time because of their correlations with all the genome properties that were tested and led to their definition as “a fundamental level of genome organization”^6^. Recently, however, investigations^7^ showed that maps of GC-rich and GC-poor isochores of all human and mouse chromosomes match maps of TADs (0.2–2 Mb in size)^8^ and maps of LADs (~0.5 Mb medium size)^9^, respectively, so providing a strong indication for a precise correlation between isochores and chromatin domains.

Very recent work^4^ led to the discovery that isochores are characterized by 3-D structures that (1) are compositionally flat in GC-poor isochores but characterized by peaks and valleys in GC-rich isochores; (2) play a crucial role in the primary formation of LADs and TADs; and (3) have widely different distributions of specific sets of tri-nucleotides such as “A/T-only” and “G/C only” tri-nucleotides in GC-rich and GC-poor isochores. Here, the wider problem concerning the general involvement of oligonucleotides in the 3D structure of the genome was approached.

## Results

### The GC profile of chromosome 21 and its connection with chromatin domains

**Figure 1:**
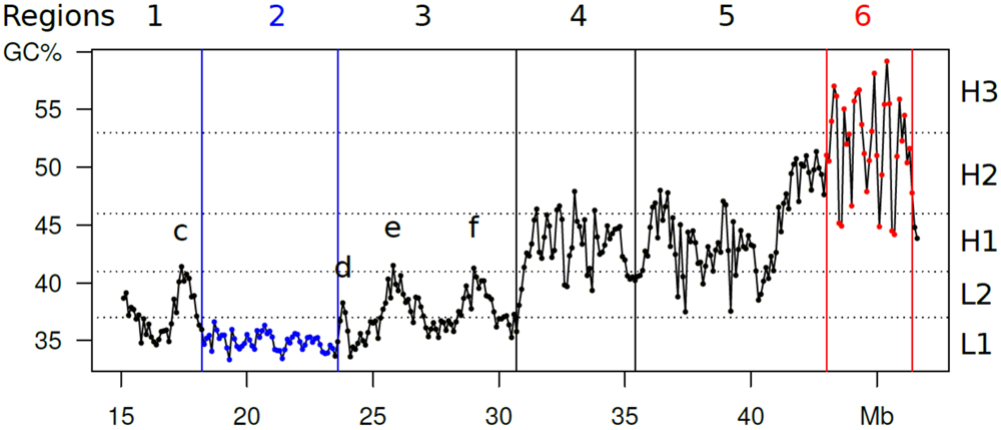
(A) Compositional profile of human chromosome 21. GC levels of 100 Kb windows (see Materials and Methods for the sequence used) are presented in a point-by-point plot. This figure shows that: region 1 comprises an L2^+^ peak (**c**); region 2 corresponds to a compositionally flat L1 isochore; region 3 comprises three L2^+^ peaks (**d–f)**; regions 4 and 5 comprise the multi-peak H1 and H2 isochores, respectively; region 6 comprises H2/H3 peaks. Black, blue and red lines separate regions 1 to 6.

The profile of chromosome 21 (as obtained using the point-by-point plot of GC levels of 100 Kb blocks^4^) is shown as a reference profile (Fig. 1). Indeed, this profile (split into several regions, numbered 1 to 6) provided the first suggestion of a 3-D structure of isochores by showing a low-heterogeneity L1 region 2 and a series of L2^+^ peaks (c to f) in regions 1 and 3, two multi-peak regions 4 and 5 corresponding to H1 and H2 isochores, respectively, and a region 6 characterized by very sharp H2/H3 peaks ranging from 40–45% to 55–60% GC. It has been recently shown^4^ that, while the GC-poor, low-heterogeneity L1 region (as well as L2^−^ regions, only represented in chromosome 21 by the “valley” isochore Y^4^, but present to much larger extents in other chromosomes) corresponds to LADs, the GC-rich, high-heterogeneity L2^+^, H1, H2, H3 peaks corresponds to TADs.

### The di-nucleotides of chromosome 21

An analysis^4^ of previous results^10,11^ (see Supplementary Fig. 1) showed that “A/T-only” and “G/C-only” di-nucleotides were the most abundant ones in GC-poor and GC-rich isochores, respectively (with AA, TT and GG, CC, respectively, reaching the highest levels); in contrast, the “mixed” dinucleotides (comprising both A or T and G or C) showed only modest differences in different isochore families. For these reasons, the profiles of the three sets of di-nucleotides were investigated separately.

**Figure 2:**
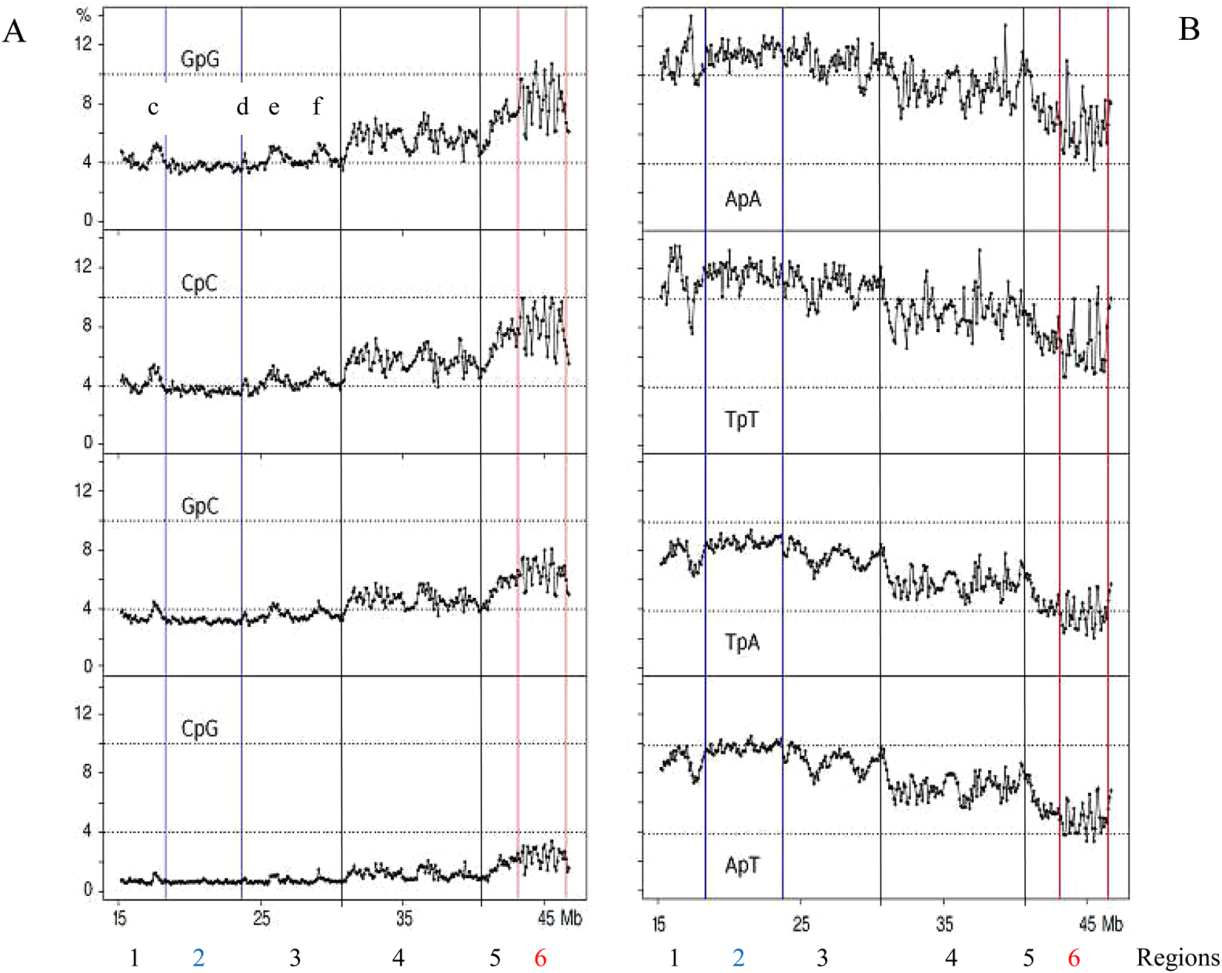
Profiles of (**A**) “G/C-only”, and (**B**) “A/T-only” di-nucleotides on human chromosome 21. Blue and red lines define regions 2 and 6, respectively. Black lines separate region 4 from the two contiguous regions 3 and 5.

The “G/C-only” di-nucleotides showed profiles (Fig. 2A) that were similar to GC profiles (Fig. 1). These profiles were characterized by a low-heterogeneity region 2 (with a ~4% level), a series of peaks c, d, e, f, in regions 1 and 3, two multi-peak regions 4 and 5 and a series of sharp GpG and CpC peaks in region 6. GpC showed a slightly lower level in region 2 and peaks covering a smaller range in region 6 compared to GpG and CpC. CpG showed the lowest levels (~1% in region 2 and small peaks in region 6), as expected from the well-known shortage of this di-nucleotide^12^.

In contrast, the “A/T-only” di-nucleotides (Fig. 2B) showed much higher levels in region 2 (~12% for ApA and TpT, with lower values, 10% and 9%, for ApT and TpA, respectively) compared to the “G/C only” di-nucleotides. In region 6, ApA and TpT showed sharp peaks although reaching lower levels compared to “G/C-only” di-nucleotides (these levels being even lower in the case of ApT and TpA) and higher levels and sharper peaks in regions 1 and 3 to 5, again especially in the case of ApA and TpT. The A/T peaks of region 3 alternated with the G/C peaks e and f. In region 5, the downward A/T slope corresponded to an upward G/C slope.

**Figure 3:**
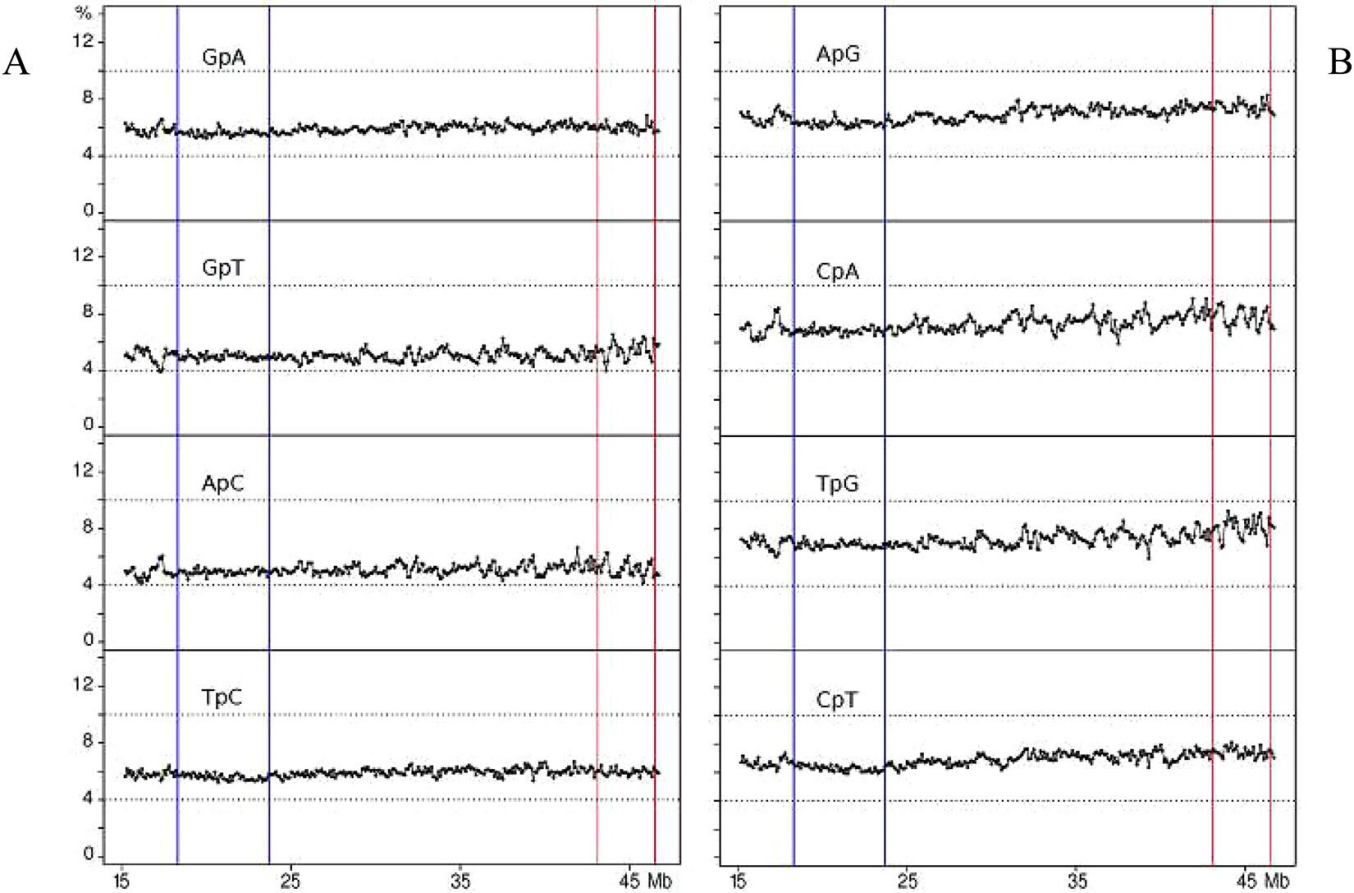
Profiles of “mixed” di-nucleotides, namely of di-nucleotides comprising both G/C and A/T.

“Mixed di-nucleotides”, namely di-nucleotides comprising both A/T’s and G/C’s, showed flat profiles with 5–7% values in region 2. Minute peaks were present in region 6 in the case of GpT, ApC, CpA and TpG (Fig. 3A,B).

### “G/C-only” and “A/T-only” tri-nucleotides

Supplementary Fig. S2 presents previous results^11^ to show that tri-nucleotides, like di-nucleotides, are characterized by frequencies in different isochore families that are different for “GC-only”, “AT-only” and “mixed” tri-nucleotides, the latter ones showing the smallest variation from L1 to H3.

**Figure 4:**
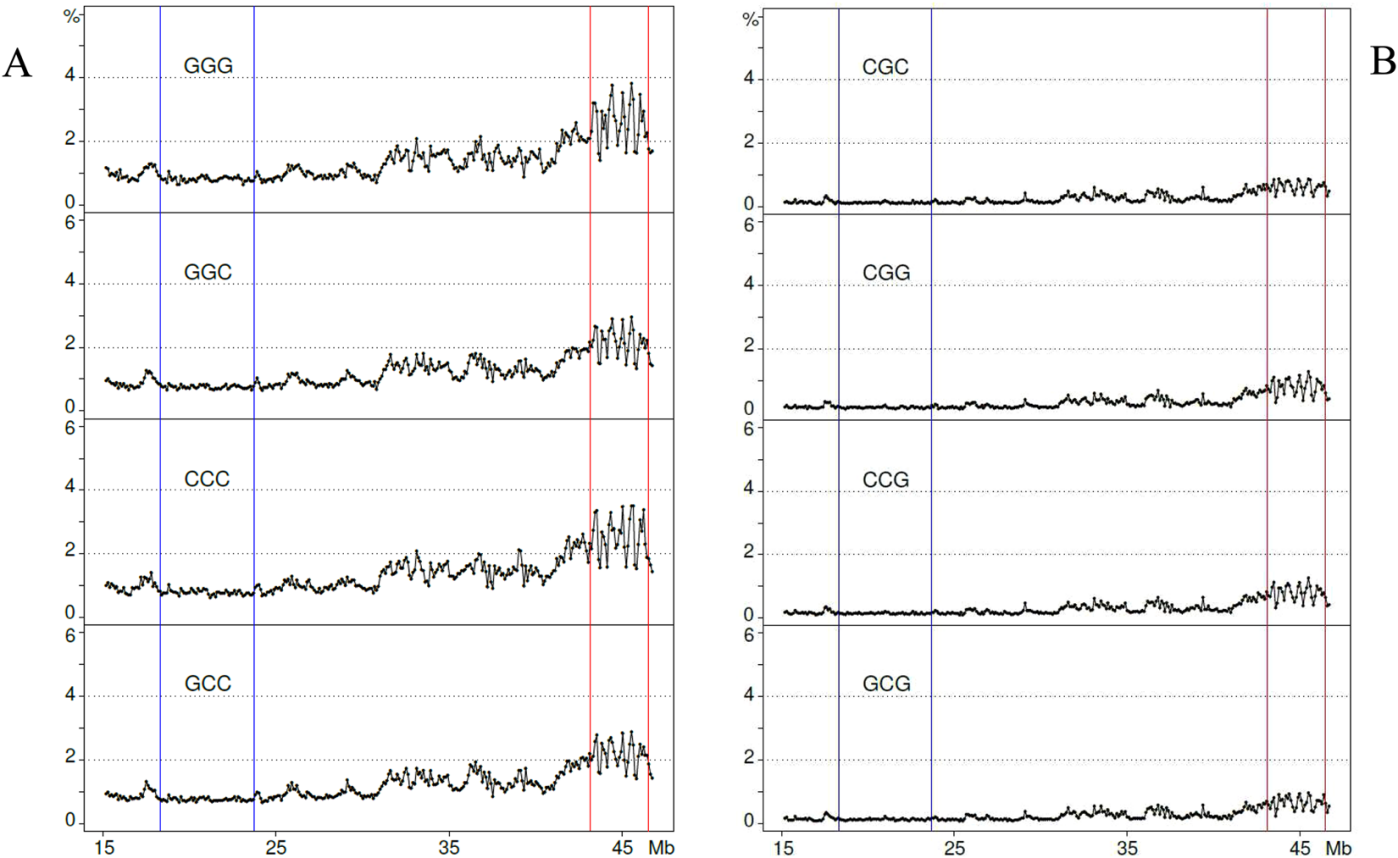
Profiles of “G/C-only” tri-nucleotides not comprising (**A**) or comprising (**B**) the CpG doublet.

Among the “G/C-only” tri-nucleotides, the GGG and CCC profiles were sharper than the GGC and GCC profiles and were similar to GC profiles in which one could detect the single peaks c to f (as well as the minute d peak just on the right side of the second blue line), the sharp peaks of region 6 and the flat low-level (~1%) region 2 (Fig. 4A). In contrast, the “G/C-only” tri-nucleotides that comprised the CpG doublet showed, as expected, very flat, very low profiles, as well as minute peaks in region 6 (Fig. 4B).

**Figure 5:**
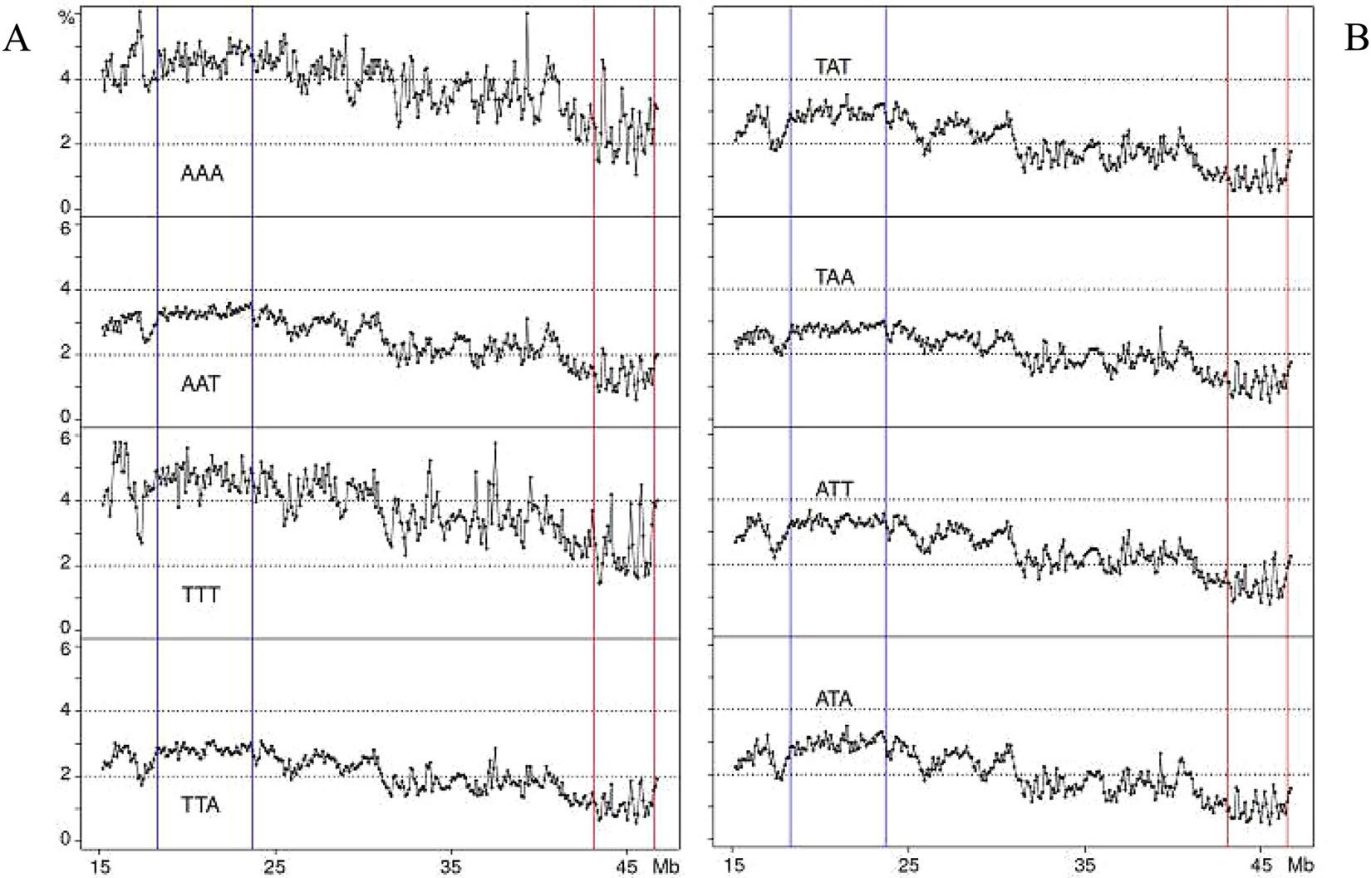
Profiles of “A/T-only” tri-nucleotides.

Among “A/T-only” tri-nucleotides, AAA and TTT showed very high levels (~5%) in region 2 and a series of peaks in regions 1 and 3–6, the peaks being extremely sharp in region 6 (Fig. 5A), whereas the other profiles (*e.g*., ATA) were lower in region 2 (~3%) and covered a smaller range in region 6 (Fig. 5B). The apparent contradiction of finding both “G/C-only” and “A/T-only” in region 6 (as well as in regions 1 and 3 to 5) could be solved by comparing in detail the two series of profiles from di- to octa-nucleotides for regions 2, 6 and 4.

### A comparison of di- to octa-As and Gs from regions 2, 6 and 4

**Figure 6:**
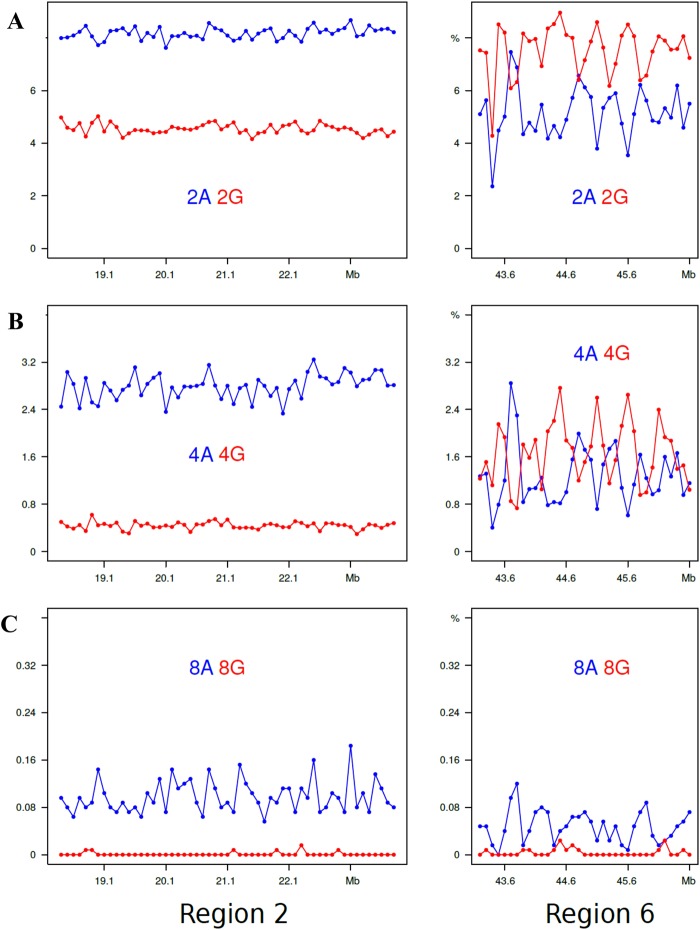
Profiles of di-, tetra-, octa-As (blue plots) and Gs (red plots) are displayed for regions 2 and 6.

**Figure 7:**
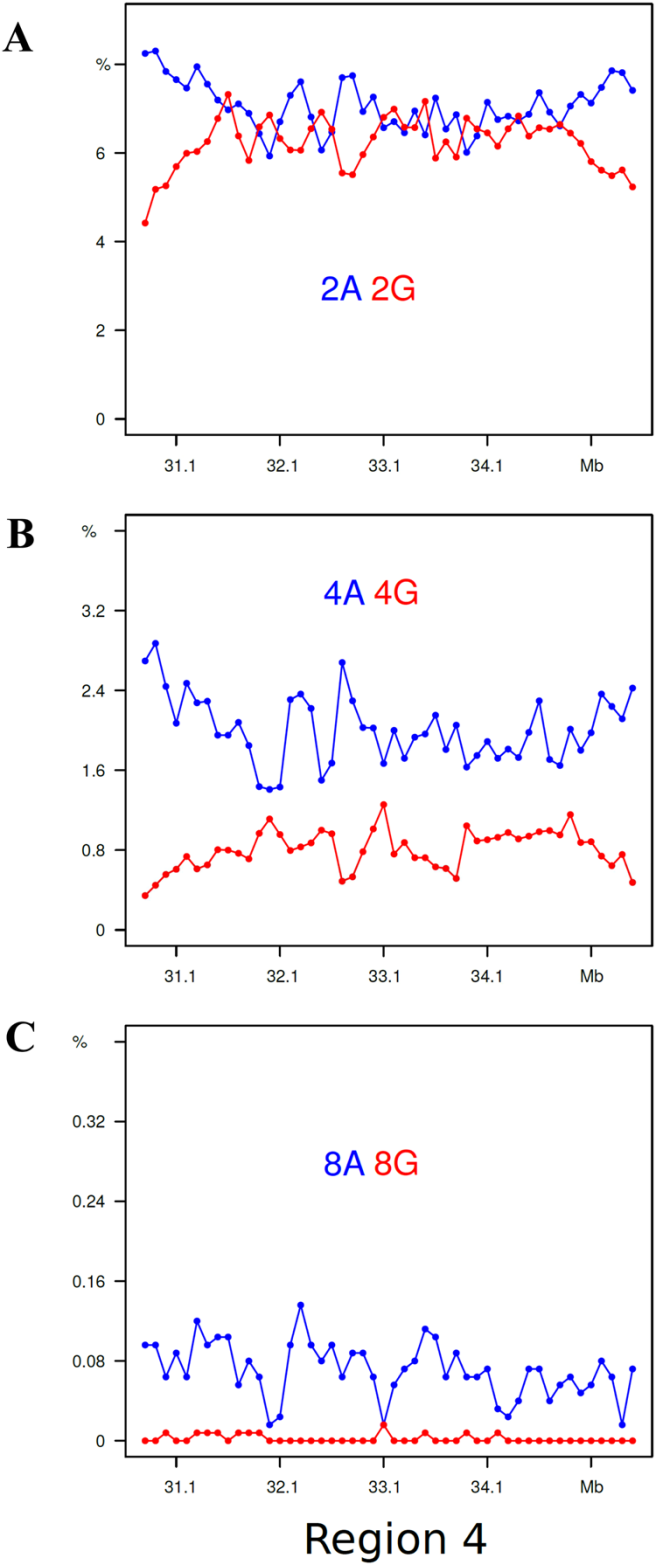
Profiles of di-, tetra-, and octa-As and Gs are displayed for region 4. Plot colors as in Fig. 6.

The results obtained for di-, tetra- and octa- A’s and G’s for regions 2 and 6 are presented in Fig. 6A,B, those for intermediate oligonucleotide sizes are shown in Supplementary Fig. S3. In region 2, the profiles are characterized, as expected, by high levels of oligo-A’s, their increasing sizes (from 2 to 8 nucleotides) having an increasingly higher level compared to the same-size oligo-G’s and being spike-shaped. In region 6, the di-nucleotides plots showed higher levels for GpG compared to ApA, a ratio which, however, progressively decreased with increasing oligonucleotide size, the octa-G profiles practically corresponding to the baseline. The most important finding was, however, that the oligo-A and oligo-G peaks were alternating, the former ones corresponding to the valleys of the latter ones. Very interestingly, this alternation was also found in the complex, multipeak region 4 (see Figs 7 and S4).

### “Mixed” tri-nucleotides

The “mixed” tri-nucleotides were shown to behave in a slightly different way according to whether they comprised two G or C and one A or T or *viceversa*, two A or T and one G or C. Indeed, the first set (see Supplementary Figs S3 and S5B) showed low values (~1–2%) in region 2 and small peaks (or, rarely, no peaks) in region 6 (as expected, tri-nucleotides comprising the CpG doublet showed extremely low values in region 2 and no peaks in region 6). The second set showed practically flat profiles (~2% or only slightly lower values) over the whole chromosome, only in some cases showing minute peaks in region 6 (see Supplementary Figs S6A and S6B).

## Discussion and Conclusions

1. This is the first analysis of a human chromosome at the level of oligonucleotides. Two choices had to be made, the first one concerning the size of oligonucleotides to be analyzed, the second one concerning the size of the chromosomal segments to be investigated. Both choices were dictated by the consideration of what was needed to demonstrate the involvement of short sequences in the 3-D structure of human DNA. The first choice was to minimize the size of oligonucleotides to di- and tri- nucleotides because this choice not only facilitated the presentation of the results, but also made possible to compare the results obtained with previous results concerning the whole human genome and generalize the conclusions (see below). The second choice, of 100 Kb, was due to our previous usage of this sequence size because 100 Kb is a plateau value under which the composition of DNA segments shows an increasing variance with decreasing size, due to the contribution of different specific sequences, such as interspersed repeated sequences^3^.
2. An important question is whether the results presented here for the di- and tri-nucleotides of chromosome 21 are generally valid for the whole human genome. This point is definitely supported by a comparison of the present results with those previously obtained for the corresponding isochore families of the human genomes^10,11^.
  In the case of “G/C-only” di-nucleotides, they are at a ~4% level in L1 region 2 (with the exception of the CpG values that are much lower) whereas peaks in H3 region 6 reach 10% for GpG and CpC. These results are in agreement with the corresponding values of L1 and H3 isochores from the whole human genome. In the case of the “AT-only” di-nucleotides, they amount to 10–12% in L1 region 2, again to the same values found for the L1 isochore family from the whole genome. If one looks at this point in more detail, one can see that the slightly lower value of ApT (~10%) and the still lower value of TpA (~9%) found for region 2 also match the corresponding values for the whole genome (see Supplementary Fig. S1). All these dinucleotides show a series of peaks in region 6 that cover a 4% to 10% range for ApA and TpT, but decrease to 4 to 6% for ApT and 3 to 5% for TpA; similar trends are shown in regions 1 and 3 to 5.
  In the case of “G/C-only” tri-nucleotides, they show low, flat profiles (~1%) in L1 region 2 and peaks in H2/H3 region 6 (with much lower values if comprising CpG doublets), as well as in region 1 and 3 to 5; these results are comparable with those of the whole genome (see Supplementary Fig. S2). “A/T-only” tri-nucleotides showed high values, 3 to 5%, in region 2 and smaller peaks in region 6, once more in agreement with the whole genome results. The slightly different behaviors of “mixed” tri-nucleotides according to whether they have 2 A/T and 1 G/C or *viceversa*, was also found in previous work on the whole genome^10,11^ (see Supplementary Fig. S2).
3. The main conclusion of the present work concerns the involvement of di- and tri-nucleotides in the topology of isochores. Indeed, “GC-only” di- and tri-nucleotides showed peaks in region 6 and low, flat levels in region 2 (the special case of CpG doublets in di- and tri-nucleotides was already mentioned). In the case of “A/T-only” di- and tri-nucleotides, levels were high in region 2, whereas peaks covering a lower range compared to “G/C-only” di- and tri-nucleotides were present in region 6 (see below). “Mixed” tri-nucleotides showed more distinct peaks in region 6 in the case of 2 G/C compared to that of 2 A/T.
  The detailed analysis of oligo-A’s and oligo-G’s in region 2 revealed that the A/G ratio shows a very strong increase from di-nucleotides to tetra-nucleotides and octa-nucleotides. In the case of region 6, the profiles are characterized by oligo-A and oligo-G peaks that alternate and show a G/A ratio decreasing from di- to tetra- and octa-nucleotides, a remarkable result in view of the high GC level of region 6. This is a very important point which also explains the results of “A/T-only” and “G/C-only” peaks in regions 1 and 3 to 5, the case of region 4 having been investigated in detail.
  Summing up, interspersed “G/C-only” di- and tri-nucleotides and oligo-Gs are distributed in increasing gradients in the peaks, whereas they have a low flat distribution in L1 region 2. In contrast, interspersed “A/T-only” di- and tri-nucleotides and oligo-As are distributed in increasing gradients in peaks that alternate with the G/C peaks, and have high levels in the L1 region 2.
4. One should now consider, as already pointed out^4^, that oligo-A’s and oligo-G’s are intrinsically stiff for different structural reasons^13^; more specifically, oligo-A’s are “intrinsically curved”^14^. Two different classes of isochores can, therefore, be distinguished from a topological viewpoint (see also ref.^4^): (1) the L1 isochores that are compositionally flat yet locally stiff because of the presence of interspersed oligo A’s and (2) the L2, H1, H2 and H3 isochores that are characterized by increasing densities of interspersed oligo-G’s alternating with increasing densities of interspersed oligo-A’s.
  A crucial point is now to consider that the features just described for the topology of GC-poor and GC-rich isochores, namely the presence of interspersed oligo-As and oligo-Gs, are strongly inhibitory to nucleosome formation^13^ and account for the different structures of chromatin domains, LADs and TADs, as already proposed^4^.
5. Two final considerations are the following. In the case of LADs (*e.g*. the L1 sequence of region 2), all di- and tri- nucleotide profiles are compositionally flat even if characterized by different levels, except for oligo-A’s that show profiles with spikes (Fig. 6). In contrast, in the case of TADs, three different situations exist according to whether di- and tri- nucleotide profiles: (1) follow the GC profiles of GC-rich isochores and, obviously contribute to them; (2) are in the form of “minute peaks”, contributing less to the GC profiles; and (3) are flat, not contributing to the GC profiles. In other words, not only oligo-A’s and oligo-G’s are involved in isochore and chromatin topology, but, also, to a lower yet definite extent, all “GC-only” and “AT-only”, as well as a large number of “mixed” di- and tri-nucleotides, as indicated by their gradients in peak regions. This opens the door to a relevant role of short sequences in the human genome (G. Bernardi in preparation).

## Materials and Methods

The compositional profile displayed in Fig. 1 concerns the DNA sequence of Human Chromosome 21 downloaded from GenBank repository, ref. NC_000021.9. The finally resulting sequence starts at nucleotide 15100001 and ends at nucleotide 46700000 of the whole chromosome; it has a length of 316 Kb and was divided into 316 non-overlapping windows of 100 Kb to perform the quantitative analyses described below.

Two different approaches were used in oligonucleotide assessments: (1) in the profiles of chr.21 all di- and tri-nucleotides were assessed (*i.e*.: for di-nucleotides, AAA will count as 2 AA); (2) in the profiles of regions 2, 4, 6, only “isolated” oligo-A’s and oligo.G’s were counted: XAAAX (X being any base other than A) will count as 1 AAA but not as 2 AA.

Homemade Perl and R scripts were written for this work.

### Nomenclature

Although TADs comprise, by definition, all the topologically associating domains, in the present context TADs indicate the chromatin domains other than LADs. The main reason for this choice is that the mechanisms of formation of the two sets of domains are different, even if based on the same DNA properties, 3-D structure of isochores and nucleosome binding.

## Electronic supplementary material

Supplementary Materials
