# Supplementary material for: The short-sequence design of DNA and its involvement in the 3-D structure of the genome
Source: Sci Rep. 2018 Dec 13;8:17820. doi: 10.1038/s41598-018-35864-9 (PMC6292894; doi:10.1038/s41598-018-35864-9)
Supplement: Supplementary file 1 — Supplementary Materials [file 41598_2018_35864_MOESM1_ESM.pdf]

Supplementary Materials

for

The short-sequence design of DNA and its involvement in the 3-D structure of the genome

Guillermo Lamolle[1], Victor Sabbia[1], Héctor Musto[1] and Giorgio Bernardi[2,3*]

[1]Laboratorio de Organización y Evolución del Genoma, Unidad Genómica Evolutiva, Facultad de Ciencias, Montevideo, Uruguay.

[2]Science Department, Roma Tre University, Viale Marconi 446, 00146 Rome, Italy.

[3]Stazione Zoologica Anton Dohrn, Villa Comunale, 80121 Naples, Italy.

*Corresponding Author

Supplementary Materials

- Supplementary Figure Legends
- Supplementary Figs S1 to S6

Numerical Tables of

1) GC profiles
2) di-nucleotide profiles
3) tri-nucleotide profiles
4) 2 to 8 oligo-A profiles for regions 2, 6 and 4
5) 2 to 8 oligo-G profiles for regions 2, 6 and 4

are available upon request.

Supplementary Figure Legends

Fig. S1. Frequencies of di-nucleotides per 100Kb DNA sequences from the five isochore families. Frequencies are calculated as percentages of the total per family (from ref.12). Permission to publish was obtained from the copyright owner.

Fig. S2. Frequencies of tri-nucleotides per 100Kb DNA sequences from the five isochore families. Frequencies are calculated as percentages of the total per family (from ref.12). Permission to publish was obtained from the copyright owner.

Fig. S3. Profiles of tri-, penta-, and hepta-As and Gs (left scales) and the GC profiles (right scales) for regions 2 and 6.

Fig. S4. Profiles of tri-, penta,- and hepta-As and Gs (left scales) for region 4.

Fig. S5. Profiles of "mixed" tri-nucleotides comprising 2 G/C and 1 A/T.

Fig. S6. Profiles of "mixed" tri-nucleotides comprising 2 A/T and 1 G/C.

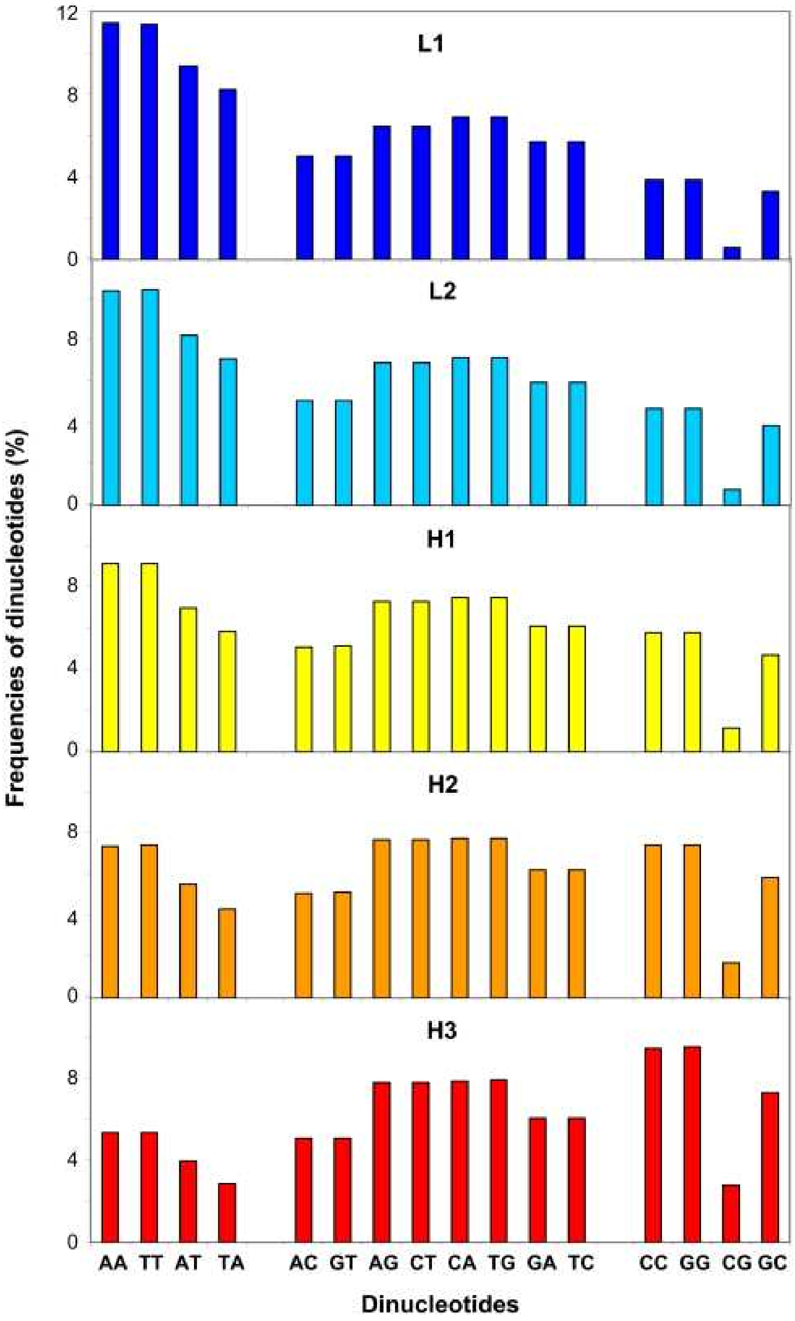

L1
L2
H1
H2
H3
12
8
4
0
Frequencies of dinucleotides (%)
AA TT AT TA AC GT AG CT CA TG GA TC CC GG CG GC
Dinucleotides

Fig.S1

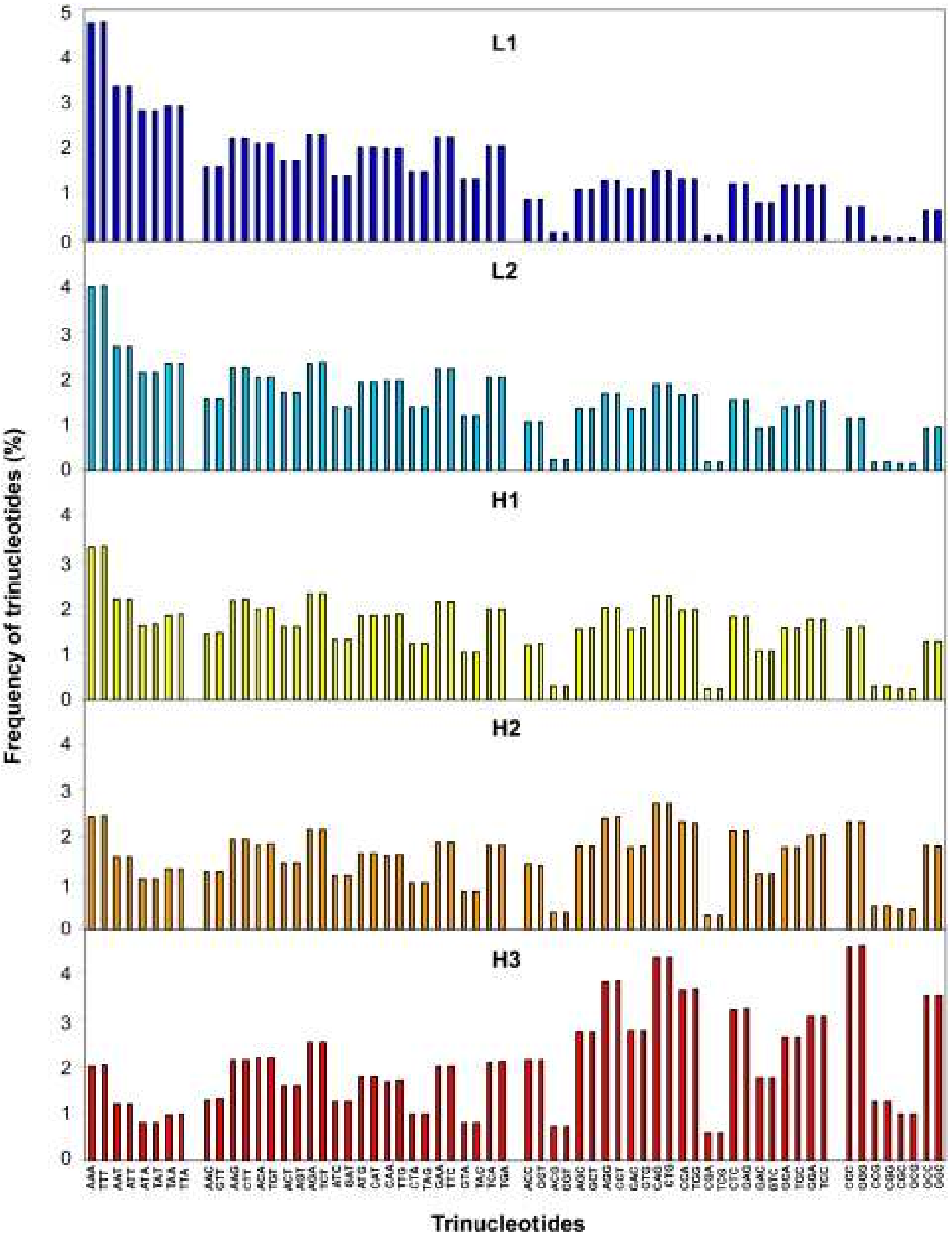

Fig.S2

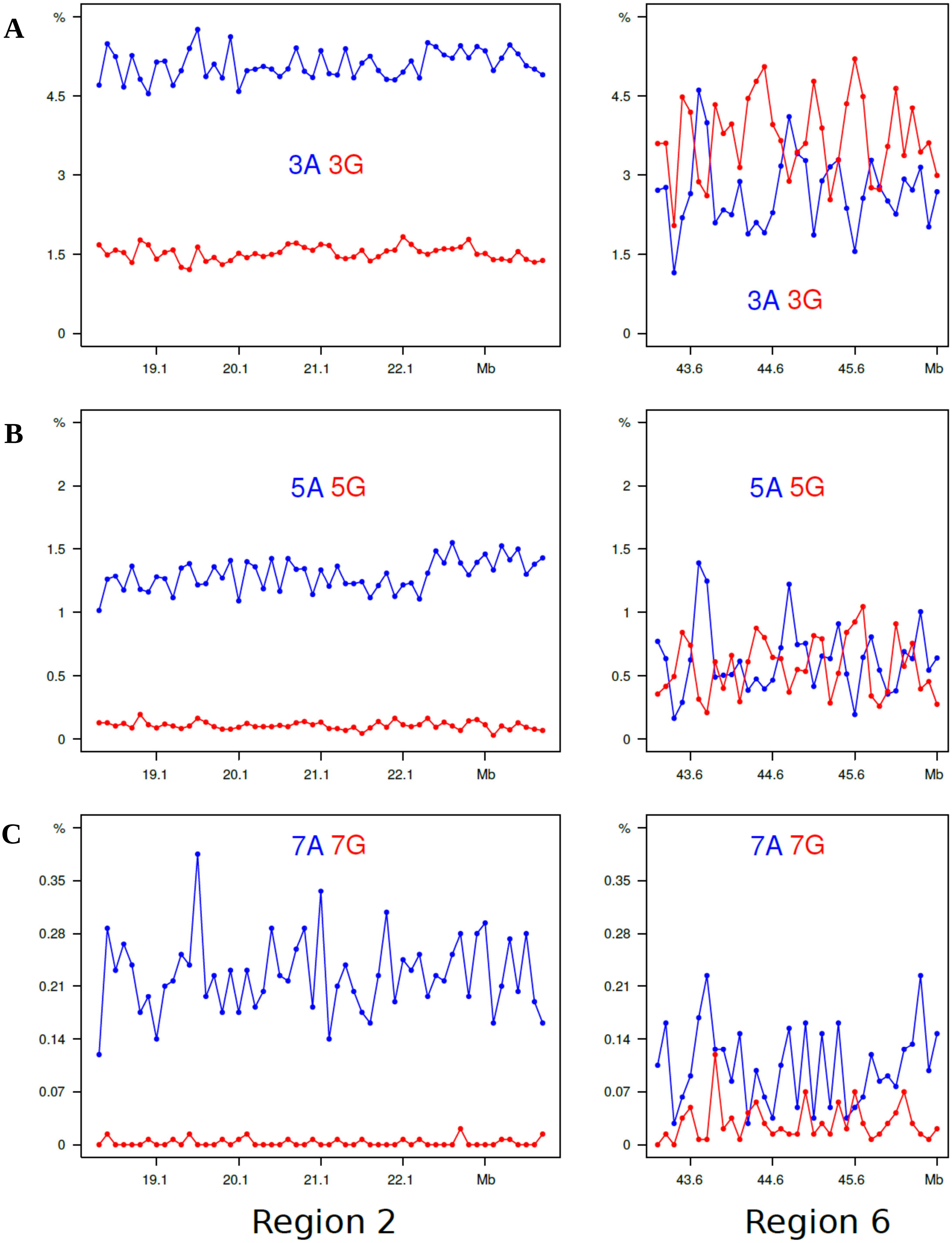
A
B
C
3A 3G
5A 5G
7A 7G
%
Mb
19.1
20.1
21.1
22.1
43.6
44.6
45.6
Region 2
Region 6

Fig.S3

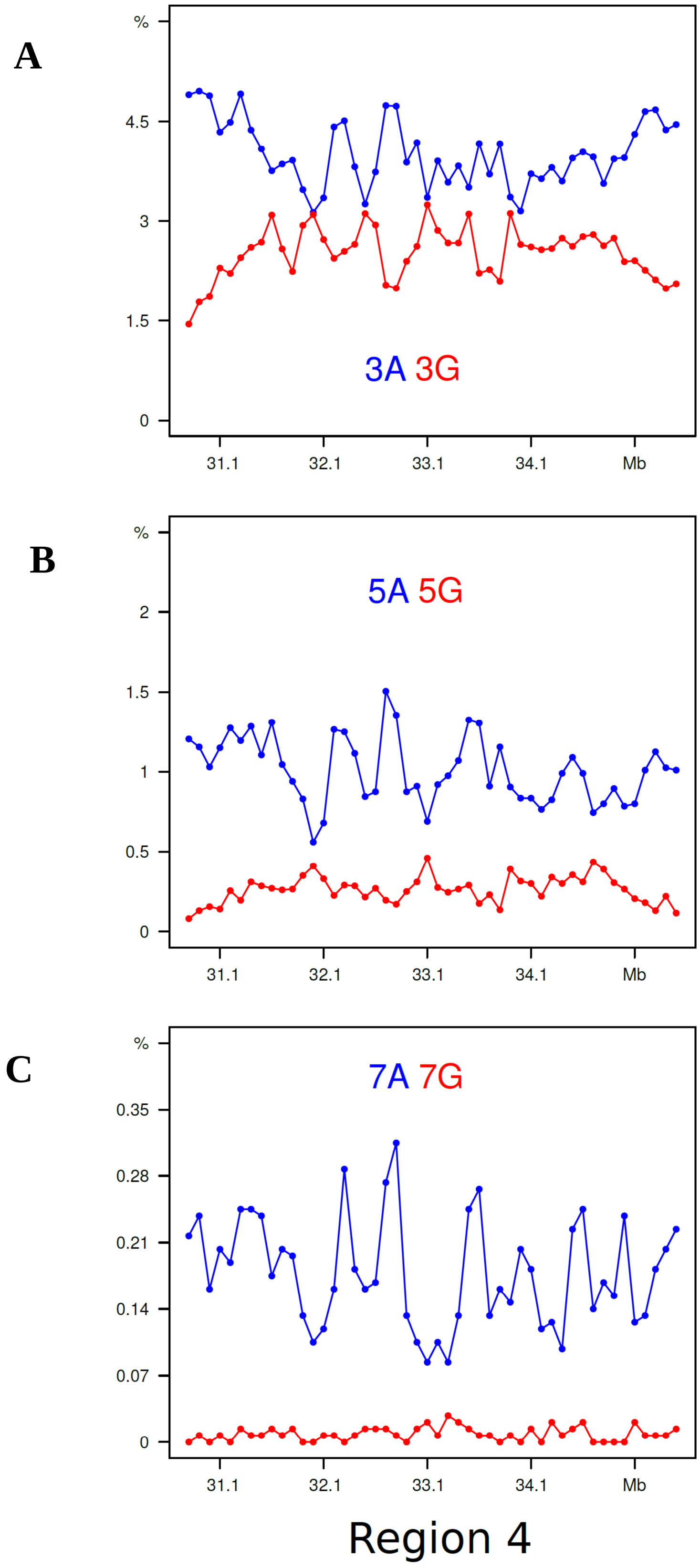

A
%
4.5
3
1.5
0
3A 3G
31.1
32.1
33.1
34.1
Mb
B
%
2
1.5
1
0.5
0
5A 5G
31.1
32.1
33.1
34.1
Mb
C
%
0.35
0.28
0.21
0.14
0.07
0
7A 7G
31.1
32.1
33.1
34.1
Mb
Region 4

Fig.S4

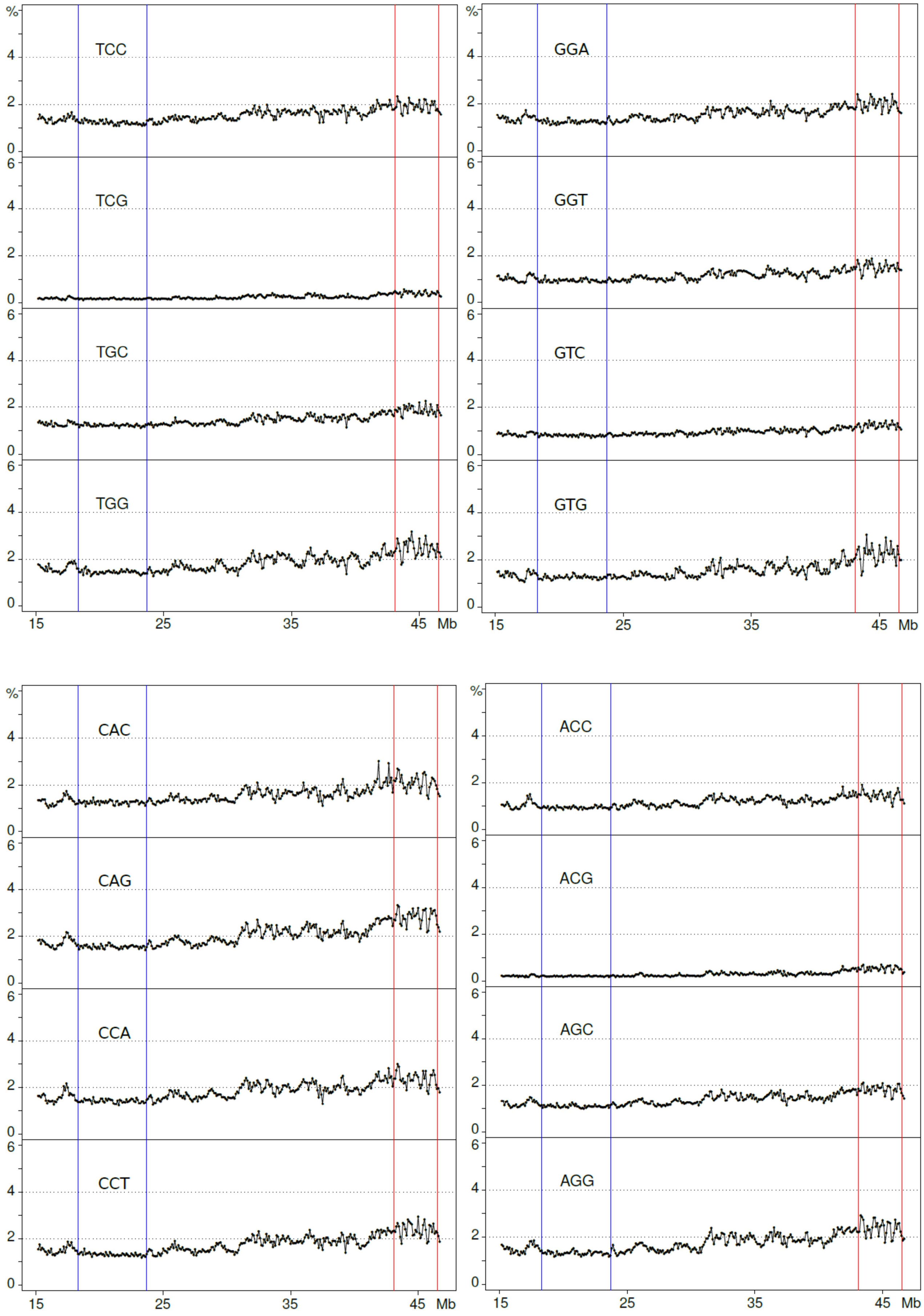

%
TCC
TCG
TGC
TGG
GGA
GGT
GTC
GTG
CAC
CAG
CCA
CCT
ACC
ACG
AGC
AGG
0
2
4
6
15
25
35
45
Mb

Fig.S5A

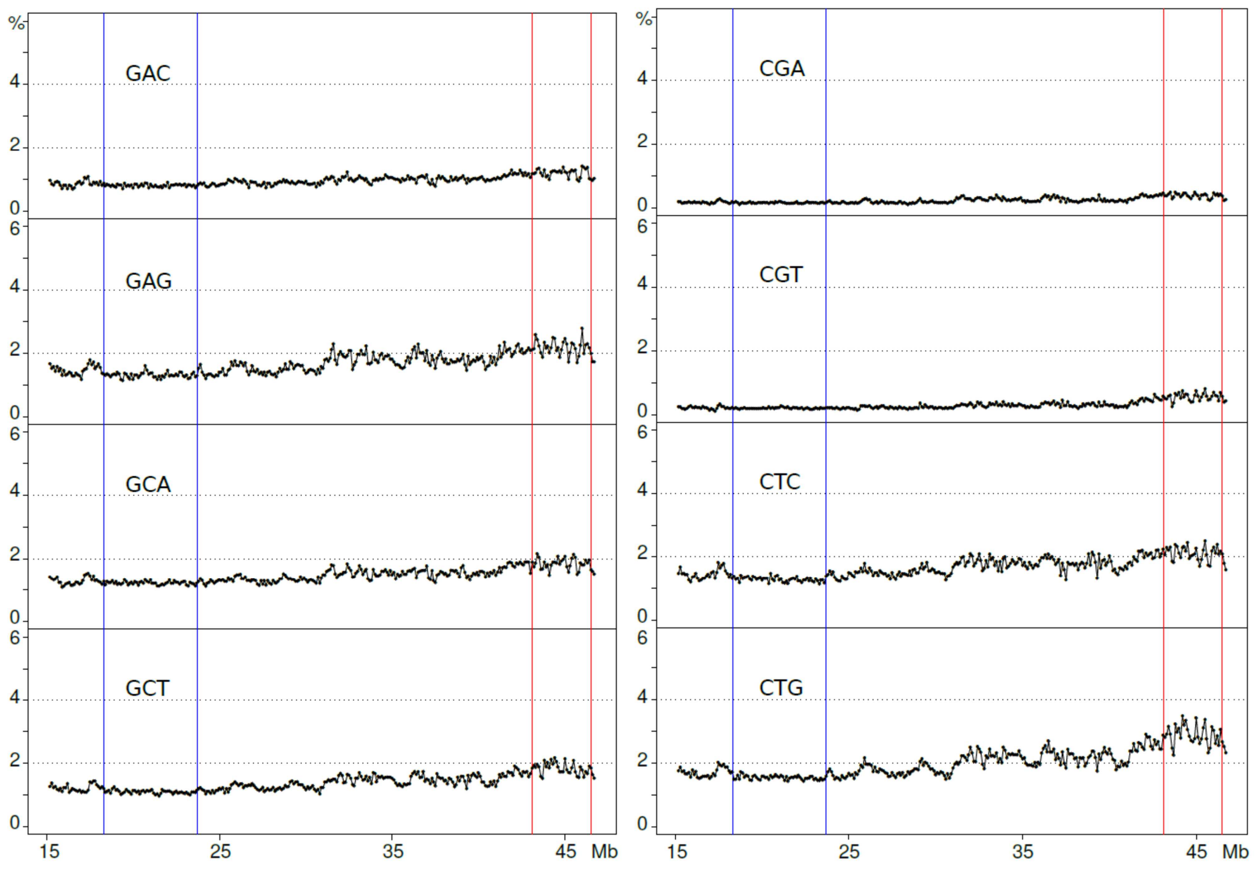

Fig.S5B

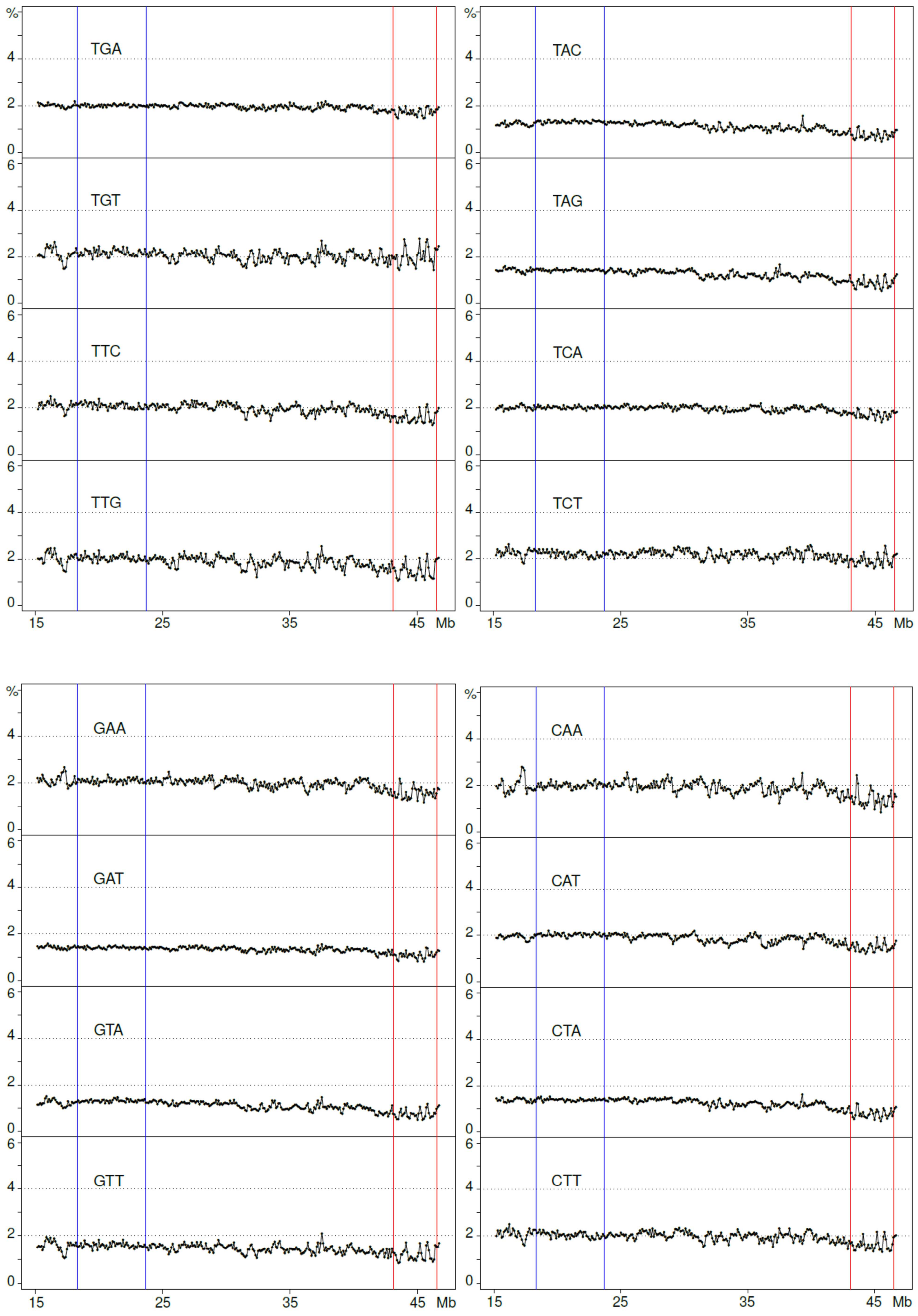

TGA
TGT
TTC
TTG
TAC
TAG
TCA
TCT
GAA
GAT
GTA
GTT
CAA
CAT
CTA
CTT
%
0
2
4
6
15
25
35
45
Mb

Fig.S6A

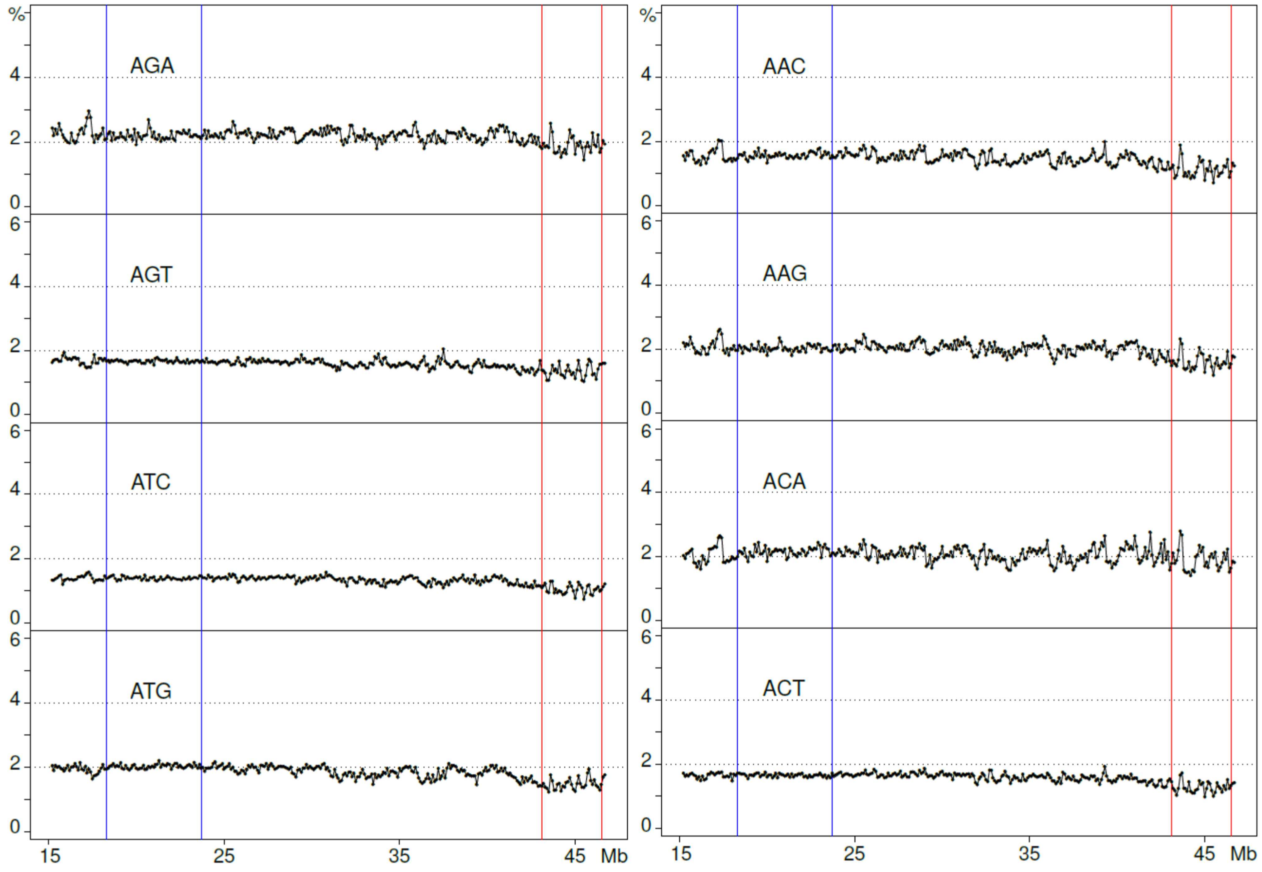

Fig.S6B
